# Analysis of aceclofenac and bovine serum albumin interaction using fluorescence quenching method for predictive, preventive, and personalized medicine

**DOI:** 10.1186/s13167-015-0047-x

**Published:** 2015-12-14

**Authors:** Sabiha Ferdowsy Koly, Sangita Paul Kundu, Shaila Kabir, Mohammad Shah Amran, Md. Zakir Sultan

**Affiliations:** Department of Pharmaceutical Chemistry, Faculty of Pharmacy, University of Dhaka, Dhaka-1000, Bangladesh; Centre for Advanced Research in Sciences, University of Dhaka, Dhaka-1000, Bangladesh

**Keywords:** Predictive, preventive, and personalized medicine, Aceclofenac, Bovine serum albumin, Fluorescence spectroscopy, Drug-protein binding, Thermodynamic parameter

## Abstract

**Background:**

The study of the interaction of a drug with plasma protein is very important because drug-protein binding plays an important role in determination of pharmacological and toxicological properties of drugs. Our study was designed to investigate the interaction between aceclofenac and bovine serum albumin (BSA) using fluorescence spectroscopy at different temperatures (298 and 308 K).

**Methods:**

Fluorescence spectroscopy was used to carry out the study. Fluorescence quenching constant was determined from Stern-Volmer equation. Van’t Hoff equation was used to determine the thermodynamic parameters such as free energy (Δ*G*), enthalpy (Δ*H*), and entropy (Δ*S*).

**Results:**

The experimental data showed that the quenching of BSA by aceclofenac was due to a formation of a BSA-aceclofenac complex with probable involvement of both tryptophan and tyrosine residues of BSA. Dynamic quenching was shown for BSA by aceclofenac at the experimental conditions. The values of thermodynamic parameters indicated that the hydrophobic forces played major roles for BSA-aceclofenac complexation. The binding number (*n*) was found to be ≈1 indicating that 1 mol of BSA bound with 1 mol of aceclofenac. The binding affinity of aceclofenac to BSA was calculated at different temperatures. It was shown that the binding constant decreased with increasing temperatures indicating that stability of the BSA-aceclofenac complex decreased with increasing temperatures.

**Conclusions:**

The interaction of aceclofenac with BSA was successfully explored using a fluorescence spectroscopic technique.

## Background

**Fig. 1:**
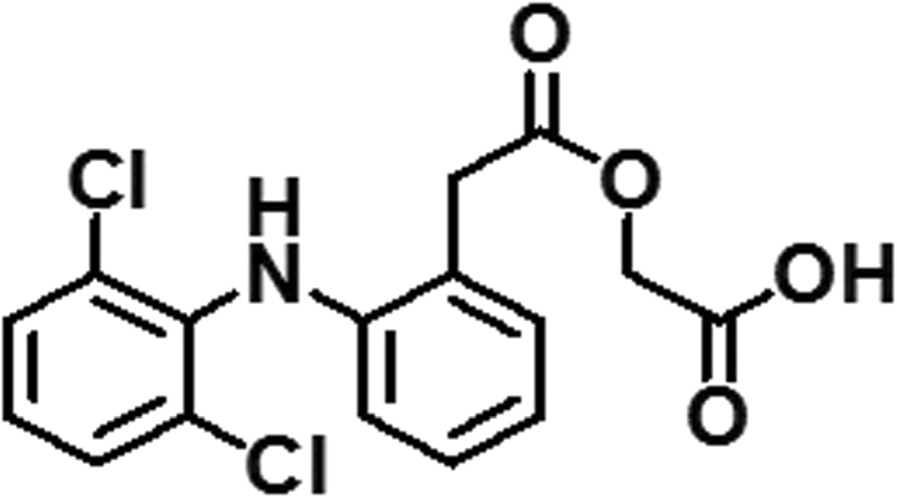
Chemical structure of aceclofenac

Plasma protein binding is a very important factor in pharmacokinetics, pharmacodynamics, and drug interaction. Drug-protein interaction greatly influences the absorption, distribution, metabolism, and excretion properties of drugs [1]. A clear perception about the features of drug-protein interaction provides insights into drug therapy and drug design. This interaction has an effect on bioavailability and toxicity [2–5]. In this study, bovine serum albumin (BSA) was used as a model protein because of its low cost, availability, and structural homology with human serum albumin [6]. Spectral methods are a powerful tool to reveal the binding of drugs with albumin at low concentrations. Binding affinities can be determined by fluorescence quenching. The fluorescence quenching technique is used to monitor the molecular interactions because of its high sensitivity, reproducibility, and relatively ease of use [7–10]. Aceclofenac (2-[(2,6-dichlorophenyl)amino]phenylacetoxyacetic acid) (Fig. 1) is a NSAID of phenyl acetic acid group derivative possessing an anti-inflammatory property which is used for the treatment of osteoarthritis, rheumatoid arthritis, and inflammatory disorders [11]. The cost-effective management of diseases has been considered as an important target of drug therapy. Considering this, we aimed to find out the possible interaction between aceclofenac and BSA for identifying the correct binding site of aceclofenac with the intension if any dose adjustment is required due to interaction [12]. To optimize the use of aceclofenac as a predictive, preventive, and personalized medicine, the study is important.

## Methods

### Drugs and chemicals

Aceclofenac was a kind gift from the Eskayef Bangladesh Ltd. BSA (fatty acid free fraction V, 96–98 %) was purchased from Sigma-Aldrich, USA. All other reagents used in the study were of analytical grade and purchased from local agents. All BSA solutions were prepared in pH 7.40 buffer solution. The buffer solution was prepared using a mixture of disodium hydrogen phosphate (Na_2_HPO_4_) and potassium dihydrogen phosphate (KH_2_PO_4_).

### Instruments

Fluorescence measurements were performed using a 1-cm quartz cell on an FL-7000 spectrofluorophotometer (Hitachi, Japan). For different temperatures, a thermostat bath (Unitronic Orbital, P-Spectra, Spain) was used.

### Sample preparation

Five milliliters of previously prepared 20 × 10^−6^ mol L^−1^ BSA in phosphate buffer of pH 7.4 was taken in each of the eight test tubes. Aceclofenac was added in different volumes to seven out of eight test tubes to have the following concentrations: (0, 20, 40, 80, 120, 160, 240, and 320) × 10^−6^ mol L^−1^, respectively. The ratios of aceclofenac and BSA ([aceclofenac]/[BSA]) in aceclofenac-BSA system of seven test tubes were 1:1, 2:1, 4:1, 6:1, 8:1, 12:1, and 16:1, respectively.

### Spectroscopic measurement

At two different temperatures (298 and 308 K), the fluorescence emission spectra for aceclofenac-BSA system were recorded at the two excitation wavelengths of BSA (280 and 293 nm). The widths of both entrance and exit slits were set to 5 nm. These emission spectra were recorded for three times for each treatment in the range of 320–460 nm for BSA at the same experimental conditions since there were no emission spectra of aceclofenac in this range.

## Results and discussion

### The interaction of aceclofenac with BSA

**Fig. 2:**
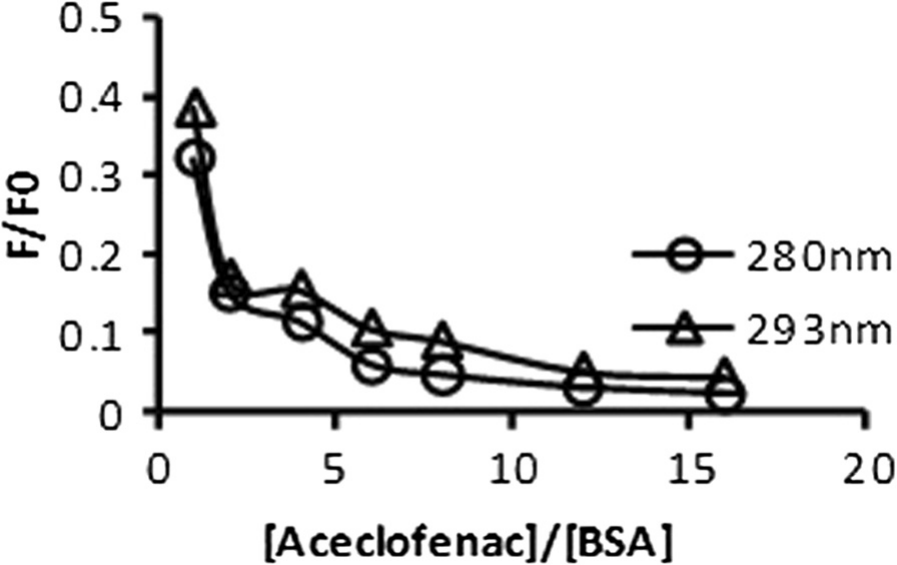
Fluorescence titration curve of BSA in the presence of aceclofenac at the excitation wavelengths of 280 and 293 nm at 298 K

If BSA is excited by appropriate wavelengths of light, all of its fluorophores (tryptophan, tyrosine, and phenylalanine) can emit fluorescence. When a 280-nm excitation wavelength is used, the fluorescence of albumin comes from both tryptophan and tyrosine residues, whereas a 293-nm wavelength only excites tryptophan residue [13]. It compared the fluorescence of BSA excited at 280 and 293 nm in the presence of aceclofenac that would determine the interactions of residues of BSA with aceclofenac. The plots *F*/*F*_0_ against [aceclofenac]/[BSA] at excitation wavelengths 280 and 293 nm were compared at 298 K, respectively. Here, *F*_0_ is the fluorescence intensity of BSA, and *F* is the fluorescence intensity of BSA in presence of aceclofenac. Figure 2 indicates that the fluorescence of BSA excited at 280 nm obviously differed from that excited at 293 nm in the presence of aceclofenac. This difference between the quenching of serum albumin fluorescence showed that both tyrosine and tryptophan residues participated in the molecular interactions between aceclofenac and BSA.

### Effect of aceclofenac on the fluorescence emission spectra of BSA

**Fig. 3:**
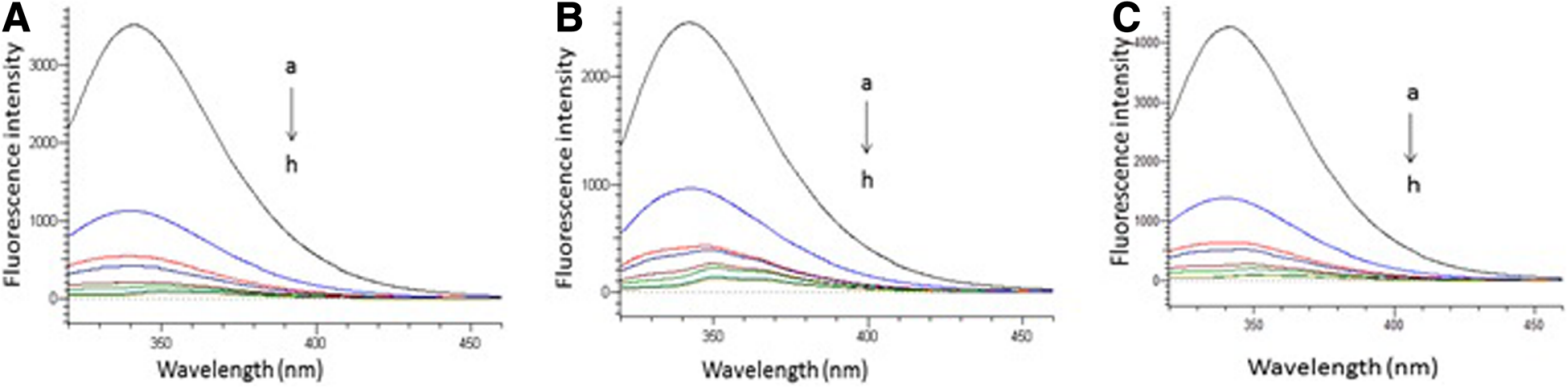
Fluorescence emission spectra of aceclofenac-BSA system at the excitation of **a** 280 nm at 298 K, **b** 293 nm at 298 K, and **c** 280 nm at 308 K. [Concentration of BSA = 0 μM; concentrations of aceclofenac from *a* to *h*: (*a*) 0, (*b*) 20, (*c*) 40, (*d*) 80, (*e*) 120, (*f*) 160, (*g*) 240, and (*h*) 320 μM]

In order to determine the effect of aceclofenac on BSA, the fluorescence emission spectra were measured at two excitation wavelengths of BSA (280 and 293 nm) at 298 K. Figure 3 shows that the fluorescence of BSA gradually decreases with the increasing concentration of aceclofenac, indicating that there was a strong interaction and energy transfer between aceclofenac and BSA at both excitation wavelengths of BSA (*λ*_max_ of BSA = 280 and 293 nm) at 298 K. As a result, there was quenching of intrinsic fluorescence of BSA but no significant shift of the emission maximum wavelength was observed.

### Fluorescence quenching analysis

Quenching is a process where the fluorescence intensity of a substance decreases in the presence of a quencher molecule [14]. The drug-protein interaction can be static and dynamic depending on the type of interaction. A variety of processes can result in quenching, such as excited state reactions, energy transfer, complex formation, and collisional quenching. The formation of a complex between the quencher and the fluorophore refers to static quenching. On the other hand, collision of the quencher and fluorophore during the excitation refers to dynamic quenching [15]. The fluorescence quenching data are usually analyzed by Stern-Volmer equation [16] which is given below:

$$ {F}_0/F=1+{K}_{\mathrm{sv}}\left[Q\right] $$

where *F*_0_ and *F* are the fluorescence intensities in the absence and presence of a quencher, [*Q*] is the quencher concentration, and *K*_sv_ is the Stern-Volmer quenching constant which indicates the strength of interaction between albumin and a quencher molecule.

**Fig. 4:**
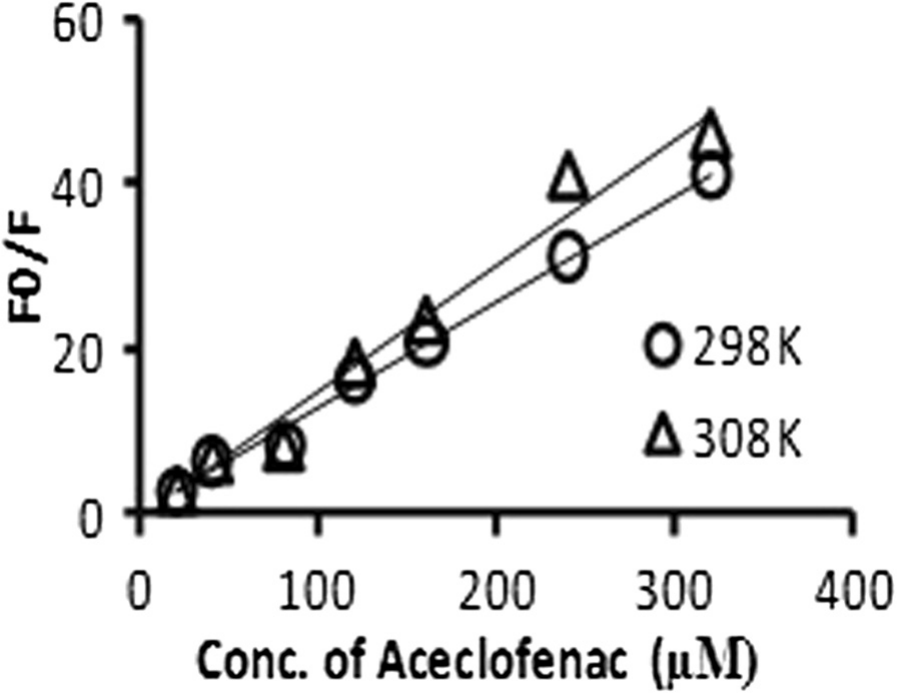
The Stern-Volmer plots for aceclofenac-BSA system at the excitation wavelength of BSA at 280 nm at two different temperatures (298 and 308 K)

Hence, this equation was applied to determine *K*_sv_ values by linear regression of a plot of *F*_0_/*F* against [*Q*]. The static quenching differs from dynamic quenching by their dependence on temperature [16]. Dynamic quenching depends upon diffusion, and higher temperatures result in larger diffusion coefficients. As a result, the Stern-Volmer quenching constants (*K*_sv_) were expected to increase with increasing temperature. In contrast, an increased temperature is likely to result in decreasing stability of complexes and thus a lower value of static quenching constants [17]. The pattern of quenching of BSA fluorescence by aceclofenac was determined by measuring the value of Stern-Volmer quenching constant (*K*_sv_) values at the excitation wavelength of BSA (280 nm) at two different temperatures (298 and 308 K). *K*_sv_ values were calculated from the slope of the plot of *F*/*F*_0_ versus the concentration of aceclofenac based on the fluorescence data (Fig. 4) at the experimental conditions.

Figure 4 displays the Stern-Volmer plots of the quenching of BSA fluorescence by aceclofenac at the excitation wavelength of BSA (280 nm) at 298 K temperature. The plots showed that within the experimental concentrations, the results were in good agreement with the Stern-Volmer equation. The plots were linear, and Stern-Volmer quenching constants were obtained from the slopes at two different temperatures. These are presented in Table 1. The Stern-Volmer quenching constant decreased with increasing temperature for static quenching while for dynamic quenching, the reverse effect was observed [18]. It was seen from Table 1 that the *K*_sv_ values increased by increasing temperature at 280 nm. So, it was found that dynamic quenching occurred for BSA in the presence of aceclofenac by increasing temperatures from 298 to 308 K.

**Table 1:** The Stern-Volmer quenching constant (*K*_sv_) for aceclofenac-BSA system at 280 nm at 298 and 308 K temperatures

Temperature (K)	Stern-Volmer quenching constant, *K*_sv_ (L mol^−1^)
298	1.27 × 10^5^
308	1.53 × 10^5^

### Determination of thermodynamic parameters and nature of binding forces

Fluorescence active substance and quencher can interact to each other through different forces like hydrophobic force, electrostatic interactions, van der Waals interactions, and hydrogen bonds.

The thermodynamic parameters were calculated in order to elucidate the interaction between the drug and BSA, which was determined from the Van’t Hoff equation [19]:

$$ \ln \kern0.5em {K}_a=-\left(\varDelta H/RT\right)+\left(\varDelta S/R\right) $$

where Δ*S* is the entropy change, Δ*H* is the enthalpy change, *R* is the universal gas constant, and *K*_a_ is the constant which is analogous to the Stern-Volmer quenching constants *K*_sv_ at the corresponding temperature.

**Fig. 5:**
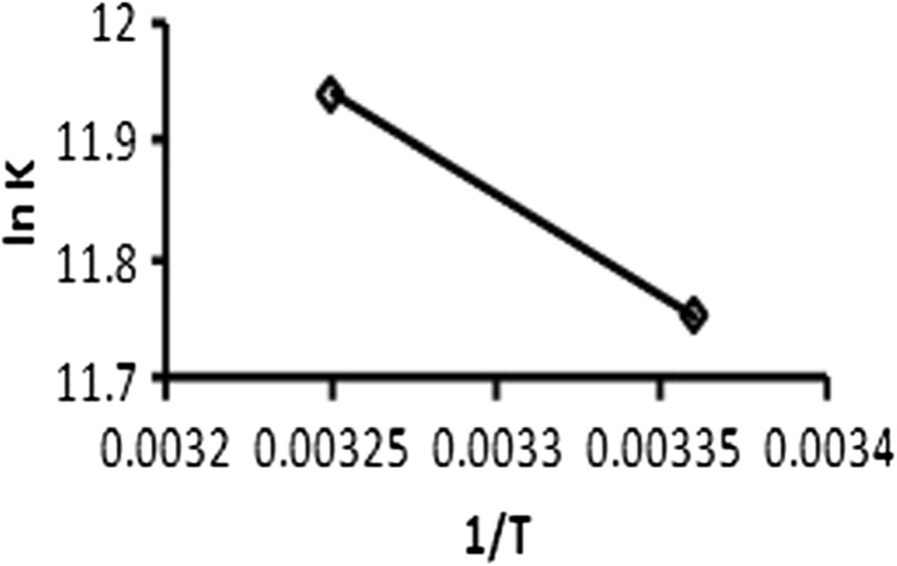
The Van’t Hoff plot for aceclofenac-BSA system at 280 nm at two different temperatures (298 and 308 K)

The enthalpy change (Δ*H*) and the entropy change (Δ*S*) can be determined from the slope and intercept of the fitted curve of ln *K*_sv_ against 1/T, respectively (Fig. 5). The free energy, Δ*G*, can be estimated from the following relationship:

$$ \varDelta G=\varDelta H\hbox{-} T\varDelta S $$

**Fig. 6:**
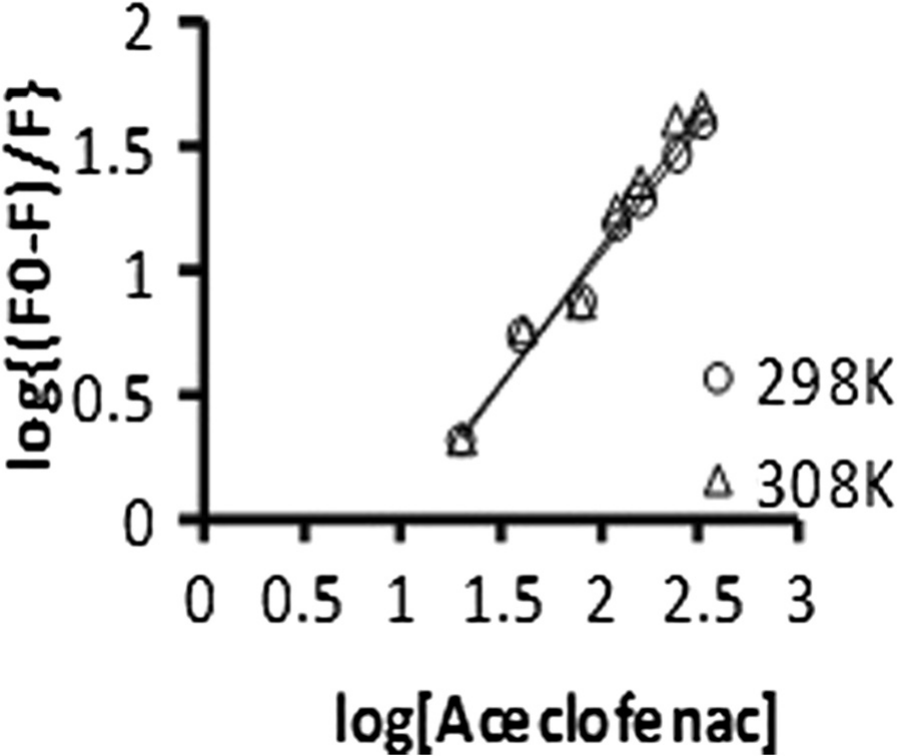
Plot for binding constant and binding points for aceclofenac-BSA at 280 nm at two different temperatures (298 and 308 K)

Table 2 indicates that the enthalpy change (Δ*H*) and the entropy change (Δ*S*) were positive and the free energy change (Δ*G*) was negative. This negative Δ*G* value indicated that the binding of aceclofenac to BSA was spontaneous. According to the views of Ross and Subramanian [20], the model of interaction between a drug and biomolecule is frequently regarded as the evidence for a hydrophobic interaction [21] because the water molecules arranged in an orderly fashion around the drug and protein establish a more random configuration. So, it can be said that hydrophobic forces are playing a major role in aceclofenac-BSA interaction at the wavelength of 280 nm and 298 and 308 K temperatures (Fig. 6).

**Table 2:** Thermodynamic parameters for aceclofenac-BSA system at 280 nm at two different temperatures (298 and 308 K)

Temperature (K)	Δ*H* (kJ mol^−1^)	Δ*S* (J mol^−1^)	Δ*G* (kJ mol^−1^)
298	14.051	144.913	−25.52
308			−30.58

### Determination of binding constant and binding points

When aceclofenac binds independently to a set of equivalent sites on BSA, the equilibrium between free and bound aceclofenac is given by the following equation [22]:

$$ \mathrm{Log}\left[\left({F}_0/F\right)/F\right]= \log\ {K}_a+n\  \log \left[Q\right] $$

where *K*_a_ is the binding constant and *n* is the number of binding sites per BSA molecule.

The values of *K*_a_ and *n* are calculated from the values of the intercept and slope of the plot of Log [(*F*_0_/*F*)/*F*] versus log [*Q*].

Table 3 shows that the values of *K*_a_ for aceclofenac and BSA decrease slightly with a rise in temperature which may indicate that the complex of BSA and aceclofenac is partly decomposed when the temperature rises.

**Table 3:** Binding constant and binding points for BSA-aceclofenac system at 280 nm excitation wavelength of BSA at two different temperatures

Temperature (K)	*K*_a_ (μM^−1^)	Number
298	0.1021	1.036
308	0.0766	1.117

The values of *n* were found to be ≈1 at both excitation wavelengths of BSA at two different temperatures. The molar ratio of the aceclofenac-BSA system at 280 nm was 1:1 which indicated that 1 mol aceclofenac binds with 1 mol of BSA.

## Conclusions

The pharmacological activity of a drug is related to protein binding. Due to change in drug-protein interaction, the activity of a drug increased or decreased. The study indicated both tryptophan and tyrosine participated in the interaction of BSA and aceclofenac. It was observed that the fluorescence quenching of BSA occurred as a result of dynamic quenching. Fluorescence quenching constants were determined using the Stern-Volmer equation and Van’t Hoff equation to provide a measure of the thermodynamic parameters Δ*G*, Δ*H*, and Δ*S*. The binding process for aceclofenac has been found spontaneous, exothermic, and entropy driven as indicated by thermodynamic analysis, and hydrophobic forces are playing a major role in the aceclofenac-BSA association. From this interaction with albumin, we can get an idea about the consequences of dose increment of this drug [23–25]. This study helps us to design the aceclofenac as a predictive, preventive, and personalized medicine.
